# Utilization of information and communication technology (ICT) among sexually transmitted disease clinics attendees with coexisting drinking problems

**DOI:** 10.1186/1756-0500-7-178

**Published:** 2014-03-26

**Authors:** Xingdi Hu, Virginia J Dodd, James C Oliverio, Robert L Cook

**Affiliations:** 1 Department of Epidemiology, University of Florida, Gainesville, FL, USA; 2 Department of Community Dentistry and Behavioral Science, University of Florida, Gainesville, FL, USA; 3 Digital Worlds Institute, University of Florida, Gainesville, FL, USA; 4 College of Medicine, University of Florida, Gainesville, FL, USA

**Keywords:** Information and communication technology (ICT), Hazardous drinking, Substance abuse, Sexually transmitted diseases (STDs), Cell phone, Email, Internet, Video games, Digital divide

## Abstract

**Background:**

Alcohol misuse remains a major risk factor for contracting sexually transmitted diseases (STDs) not typically addressed in STD clinic settings. Information and communication technology (ICT) can offer new avenues to deliver evidence-based screening and treatment for problematic drinking, however, few data exists regarding the utilization of ICT among STD clinic attendees with coexisting drinking problems. The objectives of this study are to identify STD clinics attendees with hazardous drinking, to examine socio-demographic factors associated with ICT use, and to explore individuals’ interests in engaging in ICT-based health interventions.

**Methods:**

Cross-sectional questionnaires examining alcohol consumption and ICT use were administered to 396 persons attending two non-urban STD clinics. Descriptive statistics for ICT use were calculated for both hazardous drinkers and the entire sample. Multivariable logistic regression models among hazardous drinkers identified factors significantly associated with use of each kind of ICT.

**Results:**

The mean age of the 396 participants was 25 years, 66% were females and 60% were African-Americans. One third of the sample met the criteria of hazardous drinking. ICT use in hazardous drinkers included 94% reporting having internet access at least monthly, 82% reporting having an email account, 85% reporting currently owning a cell phone, and 91% reporting use of any cell phone application. More than two thirds (73%) of hazardous drinkers were willing to play health-related video games during clinic waiting time, slightly higher than the entire sample (69%). Multivariable analyses indicated that younger age were significantly related to monthly internet use, and multifunction cell phone use, while being males and younger age were significantly associated with monthly video game playing.

**Conclusions:**

Our study demonstrates commonality of ICT use among STD clinic attendees with hazardous drinking, indicating the viability of using ICT to assist screening and behavioural intervention for alcohol misuse in STD clinic settings. Future research is needed to demonstrate the effectiveness of ICT-based health behavioural interventions in the STD clinic settings or other venues that serve populations at high risk for substance abuse, HIV or other STDs.

## Background

Hazardous alcohol consumption could impair judgement and decision-making, leading people to engage in risky sexual behaviours, such as having multiple sexual partners and/or having sex without condoms. These behaviours further lead to increased risk of sexually transmitted diseases (STDs) [1-3]. Previous studies have underscored a high rate of alcohol consumption among people attending STD clinics [4-6]. For instance, Cook et al. found a high rate of binge drinking (48.0% of men and 39.6% of women) among a sample of young adults attending a STD clinic, with 28.3% meeting the criteria for alcohol use disorder, a prevalence much higher than that found in the general population [7]. Providing screening and behavioural interventions for hazardous drinking in STD clinics offers a unique opportunity to address both hazardous drinking and risky sexual behaviours, and these interventions have the potential to reduce transmission of STDs. However, most staff members in STD clinics traditionally lack training in screening and management of problematic drinking. As a result, they do not often identify coexisting hazardous drinking and provide adequate care. Innovative methods, like information and communication technology (ICT), could provide a new avenue to deliver evidence-based screening and brief interventions for hazardous drinkers at high-risk of STDs. Such interventions could allow for consistent screening for alcohol use, are inexpensive, potentially engaging, and can minimize staff burden required for intervention delivery.

For substance abuse treatment, a number of ICT-based applications have been developed and have shown promising results [8-10]. For example, a recent meta-analysis found that computer-based treatments were effective in reducing alcohol consumption [11]. However, little is known regarding the pattern of ICT use among problematic drinkers who seek testing/treatment for STDs. Samal et al. examined internet and cellular phone use among 200 women attending an urban STD clinic and found 80% reporting internet use, and 93% having a cell phone [12]. Another study conducted in 2006 reported similar level of internet use (80%) among 251 STD clinic attendees [13]. These studies, although providing important preliminary estimates of ICT use in general STD high-risk population, did not estimate the prevalence of hazardous drinking, or characterize rate of ICT use among the hazardous drinkers, or tried to determine technology types other than cell phones or internet. Therefore, we conducted a survey among attendees of two non-urban STD clinics with the purpose of identifying those with hazardous drinking, examining their accessibility and utilization of ICT, exploring factors associated with their ICT use, and providing baseline estimates in terms of willingness to adopt ICT-based health interventions.

## Methods

From January 13, 2009 to March 10, 2009, at two public county health departments in Florida, USA, consecutive persons presenting at the STD clinics were asked to complete an anonymous, self-administered questionnaire. To ensure privacy, participants completed the questionnaire in a private room or a designated area of the clinic. To ensure anonymity, they sealed completed questionnaires in an envelope and then placed the envelope into a box with a slot on top. This study was approved by the institutional review boards of the University of Florida and the Florida Department of Health.

The first nine items of the survey assessed socio-demographic characteristics, including gender, age, race, education, employment status, marital status, and health insurance coverage. Individual alcohol consumption was measured by the Alcohol Use Disorders Identification Test (AUDIT). AUDIT has been widely used to identify patients with a wide spectrum of alcohol use disorders [14,15]. In addition to AUDIT, we also asked participants about frequency of engaging in binge drinking, defined as at least five (male) or four (female) drinks per occasion [16]. Based on both AUDIT and the binge drinking question, persons were classified as hazardous drinkers if AUDIT ≥ 8 or binge drinking was reported at least once monthly. Marijuana and other drug use were also assessed, including cocaine or crack, stimulants, anxiety medication or sedatives, prescription pain medications, hallucinogens, inhalants, and opioids.

The rest of the survey asked locally-developed questions about the type and the frequency of ICT use. “Monthly internet use” was defined as at least monthly access to internet at home, school, libraries, or work. “Daily email use” was defined as having and checking an email account at least once every day. Questions assessing cell phone use asked whether participants currently owned a cell phone, and whether they used any common cell phone functions, such as sending text messages, downloading ringtones, downloading games or taking pictures via the phone camera. Information to distinguish smartphone use from other cell phone types was not specifically asked in this survey. “Multifunction cell phone use” was defined as participants reporting both owning a cell phone and using any of above cell phone functions. “Monthly video game playing” was defined as video game playing at least once a month through various video game consoles (i.e. Nintendo Wii, XBOX360, Playstation2/3, Gameboy, and PSP).

To explore potential acceptability of using video game for health promotion, participants were asked whether they would be willing to play any health-related video game during waiting time at the STD clinic. We also assessed individuals’ privacy concern of ICT by asking whether they agreed with statements that cell phone conversations and text messaging were private. In the end, among those who reported access to these technologies, we explored their willingness to receive reminders via email or text messages about STD test results readiness.

We analysed data using the statistical package SAS 9.2. Descriptive statistics for variables of interests, including demographics and ICT use, were presented for both hazardous drinkers and the entire sample for comparison purpose. Fishers’ exact test and two-way Pearson chi-square analyses were performed to assess associations of use of each ICT modality with socio-demographic characteristics. Age was categorized into 14–24, 25–34, and 35 or older. Race was categorized into white, black, Hispanic, and other racial group, including Asian, Native Hawaiian/Pacific Islander, and American Indian/Alaskan Native. The highest level of education attained was classified into high school degree/GED or less, some college or vocational degree, and college degree or more. Marital status was categorized into three levels: being married, member of unmarried couple, and single. We generated two dichotomous variables for employment (employed/unemployed) and insurance status (insured/uninsured). Variables achieving statistical significance in the bivariate analyses were included in the multivariable logistic regression models which assessed their adjusted associations with ICT use while controlling for other covariates. Multivariable analysis was performed only among those meeting the criteria of hazardous drinking.

## Results

### Sample characteristics

Of the 489 persons approached by research staffs, eighty-one percent (n = 396) completed the questionnaires and the mean age was 25 years old (SD = 8.3). Most of the participants were females (66%, n = 258), African American (60%, n = 234), unemployed (59%, n = 231), had a high school degree or less (57%, n = 223), and were uninsured (50%, n = 199). Approximately one third of the total sample (n = 126) met the criteria for hazardous drinking. Participants were significantly (P < 0.05 or P < 0.01) more likely to report hazardous drinking if they were males, white, of older age (25+ years), had higher educational status, and/or were positive for self-reported use of marijuana or other drugs in the past year (Table 1). Forty-seven percent of the entire sample reported marijuana use in the past 12 months and 13% reported other drug use while among hazardous drinkers these proportions increased to 69% and 27%, respectively.

**Table 1:** Socio-demographic characteristics of the study population (N = 396)

		**Hazardous drinkers**^***b***^	
**Characteristics**	**Total**^***a ***^**(n = 396) N (%)**	**Yes (n = 126) N (%)**	**No (n = 264) N (%)**
Gender			
Male	134 (34)	66 (52)^*c*^	64 (25)
Female	258 (66)	60 (48)	196 (75)
Age (years)			
14-24	261 (68)	74 (60)^*d*^	185 (73)
25-34	76 (20)	33 (27)	41 (16)
35+	46 (12)	17 (13)	27 (11)
Race			
White	104 (27)	55 (44)^*c*^	49 (19)
Black	234 (60)	54 (43)	175 (67)
Hispanic	32 (8)	11 (9)	21 (8)
Other^*e*^	21 (5)	6 (5)	15 (6)
Education			
High school degree/GED or less	223 (57)	57 (45)^*c*^	161 (72)
Some college/vocational degree	131 (33)	44 (35)	86 (33)
4 year college graduate or more	38 (10)	25 (20)	13 (5)
Employment			
Unemployed	231 (59)	81 (64)	146 (56)
Employed	161 (41)	45 (36)	114 (44)
Marital status			
Married	28 (7)	7 (6)	21 (8)
Member of unmarried couple	305 (81)	98 (83)	201 (80)
Single	44 (12)	13 (11)	31 (12)
Insurance coverage			
No	199 (50)	72 (58)	124 (47)
Yes	193 (50)	53 (42)	138 (53)
Marijuana use in the past year			
No	208 (53)	39 (31)^*c*^	168 (64)
Yes	182 (47)	86 (69)	93 (36)
Other drug^*f*^ use in the past year			
No	334 (87)	90 (73)^*c*^	241 (93)
Yes	52 (13)	33 (27)	19 (7)

^*a*^Data do not always sum up to total sample size due to missing data for specific variable.

^*b*^Defined if AUDIT score > = 8 or binge drinking at least once monthly.

^*c*^p < 0.01.

^*d*^P < 0.05.

^*e*^Include Asian, Native Hawaiian/Pacific Islander, and American Indian/Alaskan Native.

^*f*^Include cocaine or crack, stimulants, anxiety medication or sedatives, prescription pain medications, hallucinogens, inhalants, and opioids.

### ICT use

Data describing ICT use were presented in Table 2. Among 126 hazardous drinkers, 94% reported internet use at least monthly, 82% reported owning an email account, and 58% reported daily email use. Eighty-five percent of hazardous drinkers currently owned a cell phone, and 91% reported use of any cell phone application: text messages (90%), taking pictures (90%), downloading ringtones (71%), music (58%) and games (45%). More than two thirds (73%) of them were willing to play health-related video games during their clinic waiting time, slightly higher than the entire sample (69%). Generally, there was no significant difference in using any modality of ICT among hazardous drinkers as compared with non-hazardous drinkers except for self-reported playing any video game at least once monthly (71% vs. 53%, P < 0.01).

**Table 2:** ICT use among persons attending two non-urban STD clinics in Florida, USA

**ICT use**	**Total**^***a***^** (n = 396) N (%)**	**Hazardous drinkers**^***b***^** (n = 126) N (%)**
Have internet access at least monthly?^*c*^	363 (93)	118 (94)
Have an email account?	306 (80)	102 (82)
Check email account at least once daily?	180 (53)	67 (58)
Currently own a cell phone?	335 (87)	105 (85)
Use cell phone applications?^*d*^	366 (94)	114 (91)
Enjoy playing a video game?	239 (69)	79 (66)
Play any video game console at least once monthly?^*e*^	230 (59)	89 (71)
Cell phone conversations are private?	330 (84)	107 (86)
Texting messages are private?	325 (84)	107 (86)
During your waiting time at clinic visits, would you be willing to play health related video games?	265 (69)	90 (73)
Would you be comfortable with receiving a reminder that you have STD results available with email or text messages?^*f*^	156 (43)	49 (41)
Would you be comfortable with receiving your actual test results with email or text messages?^*f*^	71 (19)	27 (23)

^*a*^Data do not always sum up to total sample size due to missing data for specific variable.

^*b*^Defined if AUDIT score > = 8 or binge drinking at least once monthly.

^*c*^Include internet access at home, work, school, library, or the friend’s house.

^*d*^Include downloading ringtones, music, games, sending text message, or taking pictures.

^*e*^Include Nintendo Wii, Playstation 2 or 3, Xbox360, Gameboy, and PS.

^*f*^Proportion was computed based on respondents who reported having an email account or owning any cell phone.

### Privacy concerns of ICT use

The majority of hazardous drinkers agreed that either cell phone conversations (86%) or text messages (86%) were private. Among those who reported having an email account and/or owning any cell phone, 41% reported feeling comfortable receiving email/text message reminders for their STD test results readiness. In contrast, most of them (77%) might not feel comfortable receiving test results via email/text messages.

### Factors associated with four modalities of ICT use

Table 3 showed the associations between socio-demographic characteristics and use of four modalities of ICT, including cell phone, internet, email and video games. The multivariable logistic regression model found significant associations between multifunction cell phone use and younger age (14–24 vs. 35+, Adjusted Odds Ratio (AOR) = 7.8, 95% CI (Confidence Interval): 1.8, 32.9; 25–34 vs. 35+, AOR = 6.4, 95% CI: 1.4, 29.9). Similarly, greater odds of monthly internet use were found among respondents who were younger (14–24 vs. 35+, AOR = 11.4, 95% CI: 1.4, 95.5). Higher level of education was the only variable associated with increased odds of daily email use when controlling for other socio-demographic characteristics. The multivariable model found increased odds of monthly video game playing among males and those with younger age.

**Table 3:** **Factors associated with ICT use among 126 hazardous drinkers**^**a**^

**Characteristics**	**Multifunction cell phone use**	**Monthly internet use**	**Daily email use**	**Monthly video game playing**
	**AOR (95% CI)**^***b***^	**AOR (95% CI)**	**AOR (95% CI)**	**AOR (95% CI)**
Gender				
Male	—^*c*^	—	—	**5.2 (2.2, 12.6)**
Female				Ref
Age (years)				
14-24	**7.8 (1.8, 32.9)**	**11.4 (1.4, 95.5)**	1.8 (0.4, 8.4)	**29.7 (2.9, 301.3)**
25-34	**6.4 (1.4, 29.9)**	13.6 (0.99, 186.5)	1.2 (0.2, 6.1)	**32.6 (3.0, 358.0)**
35+	Ref	Ref	Ref	Ref
Race				
White	—	—	0.1 (0.0, 1.4)	—
Black			1.3 (0.1, 36.2)	
Hispanic			1.0 (0.1, 13.1)	
Other^*d*^			Ref	
Education				
High school degree/GED or less some	Ref	Ref	Ref	—
College/vocational degree	2.4 (0.8, 7.7)	4.0 (0.4, 40.0)	**12.9 (3.7, 45.7)**	
4 Year college graduate or more	9.1 (0.9, 86.9)	2.9 (0.2, 54.7)	**14.2 (1.6, 123.8)**	
Employment				
Unemployed	Ref	—	Ref	—
Employed	2.1 (0.6, 7.2)		1.6 (0.5, 5.3)	
Marital status				
Married	—	0.3 (0.0, 16.1)	—	3.4 (0.1, 76.2)
Member of unmarried couple		0.7 (0.1, 8.9)		1.5 (0.4, 5.9)
Single		Ref		Ref
Insurance				
No	Ref	Ref	—	—
Yes	2.2 (0.7, 6.9)	7.9 (0.7, 94.7)		

^*a*^Defined if AUDIT score > = 8 or binge drinking at least once monthly.

^*b*^AOR indicates adjusted odds ratio; CI, confidence interval.

^*c*^Variables not included in multivariable logistic regression models since statistically insignificance in bivariate analysis.

^*d*^Include Asian, Native Hawaiian or Pacific Islander, and American Indian/Alaskan Native.

Significant associations (P<0.05) are highlighted in boldface.

## Discussion

Hazardous alcohol consumption was common in this sample of persons attending two non-urban STD clinics in the Southeastern United States: approximately one third of participants met hazardous drinking criteria. We found overall high rates of accessibility and utilization of various types of ICT, indicating a strong potential for the integration of ICT-based intervention into healthcare delivery in STD clinic settings. For example, information relevant to STD/HIV prevention could be administered via ICT application (i.e. web-based) to STD clinics attendees without the presence of healthcare providers. Similarly, the ICT-based program may help healthcare providers identify individuals with drinking problems and even provide brief interventions to those at increased risk of acquiring or spreading STD. In addition, most of the respondents indicated that they were willing to try health-related video games during their waiting time. While emails or text messaging may work for the purpose of notifying whether STD test results are ready, it was generally not acceptable in terms of the delivery of actual results. It is not surprising to see that age and levels of education play a major role in influencing the utilization of ICT. Thus, researchers should continuously consider these two factors in any design and development of health intervention on the basis of ICT.

The prevalent internet use found in this study is consistent with prior work based on general STD patients from urban clinical settings [12,13]. Web-based screening and brief intervention to address alcohol misuse have already been evaluated and have shown promising results in some populations. For example, a double-blind, randomized trial conducted in college students showed that the 6-week web-based brief intervention significantly reduced alcohol consumption and fewer personal problems, while these effects were not persistent after treatment ended [17].

A high rate of cell phone ownership was reported among hazardous drinkers. This finding is consistent with the results from a recent study assessing cell phone use among 266 patients receiving substance abuse treatments from 8 psychological or opioid-replacement therapy clinics [18]. Cell phone-based health interventions have been examined for up to 12 clinical areas, ranging from smoking cessation programs, pediatric vaccination reminders, to hypertension, diabetes and asthma management, whereas similar studies are lacking in the field of alcohol abuse prevention and intervention [19]. In addition, several realistic concerns have been raised regarding adoption of cell phone –based health interventions: costs of development and implementation, potential abuse of short message services (SMS) for things irrelevant to study, and lack of reimbursement for health professionals’ involvement and extra time commitment. Nevertheless, our study suggests pervasive use of cell phone among STD patients, especially for those with hazardous drinking. With the flexibility of combining voice and text messaging with interactive multimedia components, cell phone technology could increase the likelihood of successfully delivering health behavioural interventions to traditionally hard-to-reach population, such as those with alcohol misuse.

Multivariable regressions found no significant racial discrepancy in terms of ICT use. However, a “digital divide” appears to exist among people from different age groups and levels of education. Interestingly, our regression results suggest that males are much more likely to play video game than females. Health-related video games playing may provide a novel approach to deliver behavioural education and intervention. A video game-based health intervention could be offered via game consoles or computers located in clinical facilities or cell phones to reach broader populations. However, our results show that males are more likely to play video game than females, which may lead to gender disparity in access to care. Future research exploring video games as a means to implement health interventions should consider this and try to adjust the design to ensure equal responses from both genders.

Our participants appear to be interested in playing a health-related video game during their waiting time at clinic visits. Meanwhile, they also disclose great concerns of receiving STD test results via either emails or text messages. It is noted that participants may only perceive text messaging private for ordinary communication, but not private enough for delivering information as sensitive as STD results. One potential solution to secure confidentiality of text messages is to add a security code to the phone or even to the message box. This function is commonly available in smartphones and could be easily achieved by using apps designed for this purpose.

Several limitations of this study should be noted. First, our findings of ICT use represent a sample in 2009, and may not necessarily reflect the current access and utilization of ICT among general STD clinic attendees and hazardous drinkers. Second, the results of this study may not be easily generalized to other geographic areas since this study is only based on two non-urban clinics located in the Southeastern US. However, we specifically evaluated the ICT use by subgroups to allow others to compare our findings to similar settings. Finally, our study is cross-sectional, and thus we can only state that different characteristics are associated with use of certain types of ICT but not predictive.

## Conclusions

One third of persons attending two non-urban STD clinics were current hazardous drinkers, and even more used marijuana or other drugs. These findings reinforce the pressing need for intervention to address alcohol or substance abuse in STD clinic settings. Our study demonstrates commonality of ICT use among STD patients with hazardous drinking, which indicates the viability of using ICT to assist screening and behavioural intervention for alcohol misuse. Variations presented in ICT use and access across age groups, genders and education levels reflect the continued presence of a “digital divide”. Clinicians and ICT program developers interested in creating ICT-based health interventions for alcohol and substance abuse intervention should be aware of and try to address it by including multiple ICT alternatives to avoid less representation of some sub-population, like females for video games playing. More research is needed to demonstrate the acceptability and effectiveness of different types of ICT-based interventions in the STD clinic settings or other venues that serve populations at high risk for substance abuse, HIV or other STDs.

## Abbreviations

ICT: Information and communication technology; STD: Sexually transmitted disease; AUDIT: Alcohol use disorders identification test; AOR: Adjusted odds ratio; CI: Confidence interval; GED: General educational development.

## Competing interests

The authors declare that there are no competing interests in this study.

## Authors’ contributions

XH performed the data analysis and wrote the manuscript. VJD, JOC and RLC participated in the design of the study, collected the data and helped to draft the manuscript. All authors read and approved the final manuscript.
